# Explainable 3D CNNs link regional and network level disruption in early Parkinson’s MRIs to symptom progression

**DOI:** 10.1101/2025.11.17.688918

**Published:** 2025-12-03

**Authors:** Ezekiel Moroze, Basilis Zikopoulos, Arash Yazdanbakhsh

**Affiliations:** 1 Computational Neuroscience and Vision Laboratory, Department of Psychological and Brain Sciences, Boston University, Boston, MA, 02215 United States; 2 Graduate Program for Neuroscience, Boston University, Boston, MA, United States; 3 Center for Systems Neuroscience, Boston University, Boston, MA, United States; 4 Human Systems Neuroscience Laboratory, Department of Health Sciences, Program in Human Physiology, Boston University, Boston, MA, 02215 United States; 5 Department of Anatomy & Neurobiology, Boston University School of Medicine, Boston, MA, United States

## Abstract

Parkinson’s Disease (PD) is a progressive neurodegenerative disorder affecting approximately 1% of the population over 65. Clinical diagnosis typically depends on tracking gradually developing motor symptoms as the disease progresses, underscoring the need for early detection methods to aid intervention while symptoms are still minor. Inexpensive and widely available imaging modalities such as T1-weighted MRI (T1w MRI) have potential for early PD diagnosis but lack established systematic biomarkers of PD pathology. In this study, a 3D convolutional neural network (3D CNN) was trained on 100 predominately early-state PD and 100 control T1w MRIs from Parkinson’s Progression Markers Initiative (PPMI), achieving a classification accuracy of 84.5%. Misclassified subjects were majority unmedicated and particularly early PD (< 3 years since first symptoms). To interrogate the biological basis behind the model’s decisions, novel explainability methods were applied to generate regional saliency maps from both PD and control classifications. Regional saliency across subjects correlated best with cognitive and motor scores in nigrostriatal and other subcortical regions, as well as in temporal and insular cortices, indicating changes in these areas were best connected with symptom progression. The model was also sensitive to changes in the left frontal cortex across many subjects, which exhibited the greatest raw saliency magnitude. Pairwise saliency correlation was most pronounced between areas within the same functional network, suggesting the CNN was sensitive to network level changes in structural MRI. These findings demonstrate the potential of explainable 3D CNNs to identify network and regional biomarkers of early PD from T1w MRI.

## Introduction:

Parkinson’s Disease (PD) is a neurodegenerative disorder which affects between 1–2% of the population over the age of 65^1,2^. Dysfunction and eventual death of dopaminergic neurons in the substantia nigra pars compacta (SNc) and associated areas is thought to be a primary neuropathological feature of early PD^3,4^. This atrophy drives a set of motor symptoms characteristic of PD, namely bradykinesia (slowness of movement), tremors, and rigidity^4,5^, and is further associated with decreased cognitive performance, changes in mood, decision-making, and sleep patterns^5–8^. SNc atrophy cannot be reliably measured from widely available T1-weighted (T1w) MRI, making it an unreliable tool for diagnosis^9,10^. Instead, PD diagnosis centers around patient history and motor symptoms but may be supported by more specific imaging modalities like dopamine transporter (DAT) SPECT^4,11,12^.

Due to difficulty visualizing atrophy in dopaminergic regions of interest (ROIs), widely available T1w MRI is limited as a diagnostic tool for PD^9^. However, analyses of T1 MRIs have shown that cortical thickness changes in PD relative to healthy control in a way that correlates with cognitive decline^7,13,14^. Other T1 MRI cortical thickness studies have observed a general spatial profile of cortical degeneration along PD progression, with thinning of frontal regions and the insula in the left hemisphere occurring in subjects less than 10 years from experiencing their first symptoms, and bilateral, widespread atrophy after 10 years^15^. These findings indicate that T1w MRIs contain features of PD pathology across the disease’s time course; given their worldwide availability, it would be advantageous to use novel big data approaches like deep learning to fully analyze how T1w MRIs show effects of PD.

T1w MRIs have been used to train 2D convolutional networks (CNNs) to distinguish PD vs control (CT) at accuracies up to 70%, indicating there are discriminative features identifiable by deep learning models in structural imaging^16^. 2D CNNs must be trained on MRI slices, meaning the models are unable to consider continuity across the entire MRI volume. Advanced 3D CNNs bypass this constraint by accounting for context across the entire brain in all 3 dimensions, granting an enhanced ability to reliably recognize subtle spatial patterns and make accurate classifications when provided ample data for training. While 3D models have the advantage in processing all features of MRIs, they require more training data than 2D because they cannot treat sections from a single MRI as multiple datapoints^17^. Due to this limitation, sourcing enough images to train a 3D CNN for medical imaging tasks has been historically difficult^17^. Fortunately, the recent creation of PPMI (Parkinson’s Progressive Markers Initiative) has provided access to large, high quality multi-center imaging datasets necessary to train effective 3D CNNs^18–21^.

One such model is the 3D CNN proposed in Chakraborty et al 2020. The 3D model outperformed 2D equivalents, boasting a high average accuracy of 92.75% when classifying PD versus control on about 400 MRIs from PPMI^22^. As deep learning applications become more accurate and common in medical imaging analysis, it is critical to extensively analyze these complex models to unlock explanations for their classifications and avoid the “black box” pitfall commonly associated with AI^23^. Making high quality explainability a priority has shown to increase trust among clinicians, a necessity if these tools ever enter clinical settings^24^.

To address the issue of explainability in models, we employed a novel combination of explainability methods unlocked by our proposed 3D CNN, which was able to discriminate early PD (<10 years since experiencing first symptoms) from control at an accuracy of 84.5% using 3T T1w MRIs sourced from PPMI. By combining a gradient based visualization of network classifications with an anatomical atlas, we generated regional saliency maps indicating which parts of each MRI contributed most to their classification as PD or control. Correlation between saliences of different regions implicated the nigrostriatal pathway and other canonical functional networks involved in PD, showing connective dynamics can be extracted from our 3D CNN’s processing of structural data. By establishing saliency score correlations with real subject motor scores, cognitive scores, duration of disease, and medication use, we simultaneously provided strong evidence connecting our network’s explainable output with subject data provided by PPMI and identified specific regions associated with symptoms and interventions in PD.

## Methods:

### Dataset Curation and Preprocessing:

**Figure 1:**
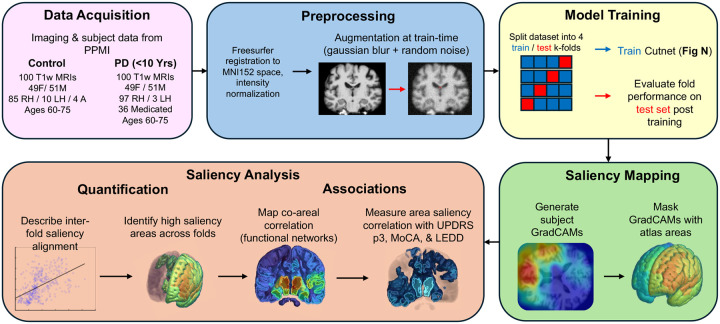
Graphical summary of study. Flow chart detailing how data was acquired and processed before training the model and how saliency data was computed and interpreted in analysis.

3D Parkinson’s MRIs and subject data were sourced from the Parkinson’s Progression Marker’s Initiative (PPMI). The PD dataset was composed of 100 3T T1-weighted MRIs with 51 male, 49 female, sampled from the age range 60–75 (mean: 67.02, standard deviation: 0.44) with less than 10 years since experiencing first PD symptoms (mean: 2.32 years, standard deviation: 1.04 years). 97 PD subjects were right-handed and 3 were left-handed, with 36 taking levodopa or an equivalent dopamine precursor at the time of imaging. The control dataset was formed of 100 3T T1-weighted MRIs with 51 male, 49 female, sampled from the same age range (mean: 66.76, SD: 0.4221). 85 subjects were right-handed, 10 left-handed, four ambidextrous, and one missing data. All MRIs were skull-stripped, intensity normalized, rescaled to 0–255 intensity range, then registered to the MNI152 atlas space using Freesurfer v7.3.2. Subject Movement Disorders Society Unified Parkinson’s Disease Rating Scale Part 3 scores (MDS-UPDRS P3, or just UPDRS P3), Montreal Cognitive Assessment scores (MoCA), and Levodopa equivalent daily dose (LEDD) were sourced from PPMI at the nearest available time point to their corresponding MRI in the imaging set. Time since symptom onset in years (YO) was calculated by taking the difference between the scan date and subject reported date of symptom onset. Details regarding data acquisition and preprocessing are also summarized in Fig 1.

### Network Architecture:

**Figure 2:**
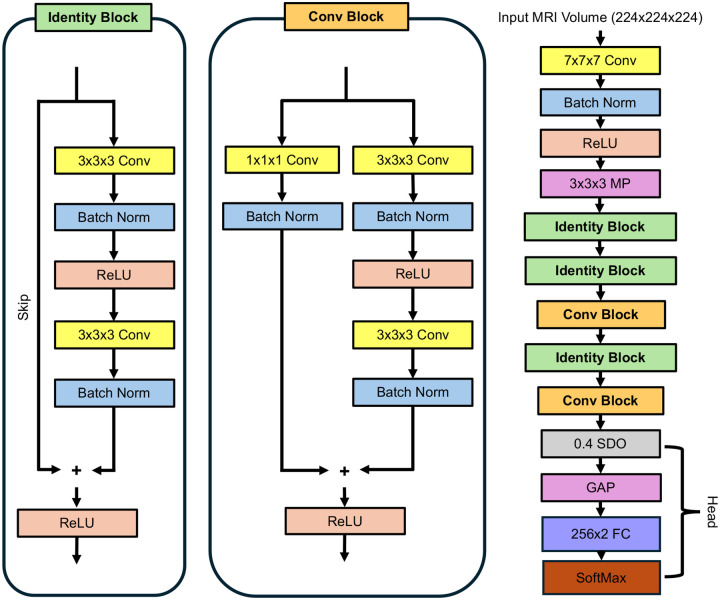
Cutnet classifier architecture. The 3D CNN accepted 224×224×224 MRI images as input, which are rescaled to 0–1 by the input layer. Cutnet is built from two different residual blocks as defined in Resnet architecture scheme, defined here as the identity block and conv block^28^. Classification is handled by a head segment containing a spatial dropout (SDO) with probability 0.4, a global average pooling layer (GAP), fully connected layer (FC), and finally a SoftMax/classification layer which outputs final class scores. Other abbreviations: rectified linear unit (ReLU); max pooling (MP).

A Resnet-18 classifier pretrained on ImageNet (a dataset with 1.2 million images belonging to 1000 categories) was used as a base for the model. We selected a pretrained model to take advantage of a modified transfer learning paradigm, where our model was first pretrained in 2D on the large ImageNet dataset vaguely related to PD detection before being “transferred” to the PD vs CT task. This protocol has shown to boost performance over naïve networks in many classification domains, including medical imaging^23,25^. To enable classification of MRI volumes instead of 2D sections while taking advantage of transfer learning, its weights were replicated in the Z-dimension to make 2D layers into 3D layers. The 3D Resnet-18 model was shortened to 12 learnable layers to reduce the number of parameters in the model and reduce the model’s ability to memorize our dataset and overfit (Referred to as “Cutnet” with 4.7M learnable parameters vs. 3D Resnet-18’s 33.2M). Prior to training, the sequence with the fully connected layer, SoftMax, and classification layer (known as the classification head) was supplemented with a spatial dropout layer of probability 0.4 to increase generalizability during training^26^. The pretrained fully connected layer was replaced with 256×2 fully connected layer for binary classification of PD vs control. Bias was initialized as zeros and weights initialized via Glorot^27^. The learning rate scalar of the fully connected layer was set to be 5 to limit relative weight change of pre-trained layers compared to the random weights of the replaced fully connected layer. See Fig 2 for a schematic of the model architecture.

### Training & Reducing Data Bias (Harmonization):

Following data preprocessing, data was randomly divided into 4 folds (150 training and 50 testing volumes per fold) such that each MRI volume was used as testing once and training 3 times. K-fold cross validation enabled maximum usage of our limited data while using each volume as testing once to better assess the performance of the model across all data. Base Cutnet networks with pretrained weights were trained in MATLAB 2023b on each of the 4 folds of data. Each fold used the same hyperparameter set as determined by a grid search: ADAM optimizer, mini batch size of 10, 20 epochs, static learning rate of 3.0 * 10^−4^, shuffle data each epoch, and L2 regularization of 5.0 * 10^−4^. The input layer rescaled input data between [0, 1] to assist gradient regularization. Two random augmentations were deployed on the training data per epoch. We utilized a 16×16×16 spatial gaussian blurring with a uniformly sampled random standard deviation between 0 and 1.5, followed by a 2% drop in overall intensity (to preserve range between 0–255) and addition of a noise mask of uniformly sampled random intensities between 0 and 5 over the entire volume.

**Figure 3:**
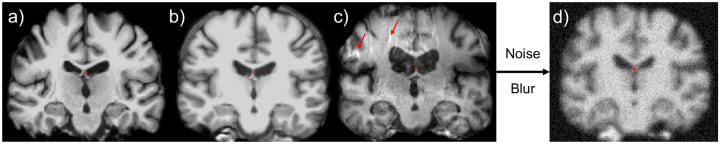
Bias remaining in data post Freesurfer normalization and example of augmentation. All MRI volumes shown were sampled from PD category after Freesurfer intensity and spatial normalization. Panel a) represents a well processed MRI, with clear distinction between white and gray matter, and the majority white matter normalized to one intensity. b) is blurrier, and c) contains more noise and artifacts (red arrows). d) A volume post augmentation, making artifacts more difficult for human observers and the network alike to parse.

Random augmentations were deployed because the model still tended to overfit during training even with the lower parameter Cutnet architecture. Even after preprocessing, manual examination revealed differing levels of blur and artifacts/noise across the data (Fig 3). Given the limited size of the dataset, it was possible imbalances in data/preprocessing quality across CT and PD classes created bias between classes that a high parameter network could be sensitive to. Thus, our augmentation procedure was designed to harmonize by injecting randomness into the same domains that the MRI dataset contained non-class-specific noise. This protocol was able to boost network accuracy by about 5% across all folds and thus was successful in making the network less sensitive to these non-PD-correspondent biases. Some of these differences in MRI quality may have been a consequence of PPMI being a multi-center dataset, with images being acquired from multiple brands of MRI machines. It has been found that CNNs can distinguish between MRI data taken from multiple centers/scanner brands with no other obvious differences^29,30^.

### GradCAM Generation, Atlasing & Masking:

**Figure 4:**
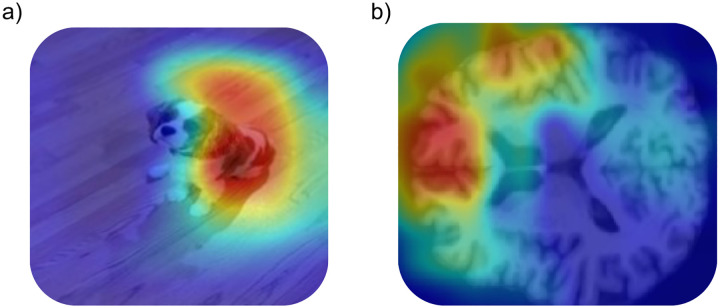
Examples of GradCAM saliency maps for different types of data. a): the GradCAM from class “English Springer Spaniel” for an image of a dog, classified by a Resnet-18 trained on ImageNet. The GradCAM saliency map shows the dog’s body and ear are salient features. b): an axial slice from a PD subject’s GradCAM saliency map for class “PD”. Hotter regions can be interpreted similarly: the warmer parts of the brain are most informative to this subject’s PD classification.

GradCAM saliency maps provided voxel-wise salience (or importance) maps across the whole subject MRI volume, in essence showing the weighting of each voxel for each subject’s classification outcome (Fig 4).

GradCAM works by taking the partial derivative of the loss for a given MRI with respect to a compressed feature-map, resulting in a channel weighting that indicates class specific importance:

(1) $$W_{c}^{h}=\frac{1}{N}\sum_{x}\sum_{y}\sum_{z}\frac{{\partial y}^{h}}{{\partial A}_{x.y.z}^{c}}$$

Where *W* is a weight corresponding to a specific channel *c* of the feature-map for binary class *h*. *W* is a summation of partial differential of class loss y^h^ with respect to a spatial feature $A_{x.y.z}^{c}$ across all 3 dimensions. The final weight is normalized by *N*, the number of voxels in the feature-map. Finally, these weights are then half-rectified and up sampled as follows to get a voxel wise gradient-weighted class activation map *M* in the input space (known as the GradCAM saliency map)^31,32^:

(2) $$M=\text{ReLU}\left(\sum_{c}A^{c}W_{c}^{h}\right)$$

As a result of the 4-fold training procedure, each subject in the dataset was used as training three times and as testing exactly once. Thus, each subject had a GradCAM saliency map generated from the network fold where they were in the unseen testing set, ensuring our saliency dataset was composed entirely of hold-out data to mitigate influence of overfitting. Once saliency maps were generated for each subject, saliency scores for each of the 414 areas from the Julich-Brain Probabilistic Atlas v3.1 were calculated by taking an element-wise weighted average of each brain area’s mask and a subject’s GradCAM saliency volume^33^. What resulted was a subject-specific vector containing a saliency value for each of the 414 atlas areas. This was done for each of the subjects in the study. The Siibra Python package was used to extract all probabilistic Julich atlas area names and masks in MNI152 space (note: probabilistic atlas areas are demarcated by “statistical” keyword in Siibra)^34^. A full list of atlas areas is available in Supp Data 1.

### Inter-fold Comparison and Validation:

**Figure 5:**
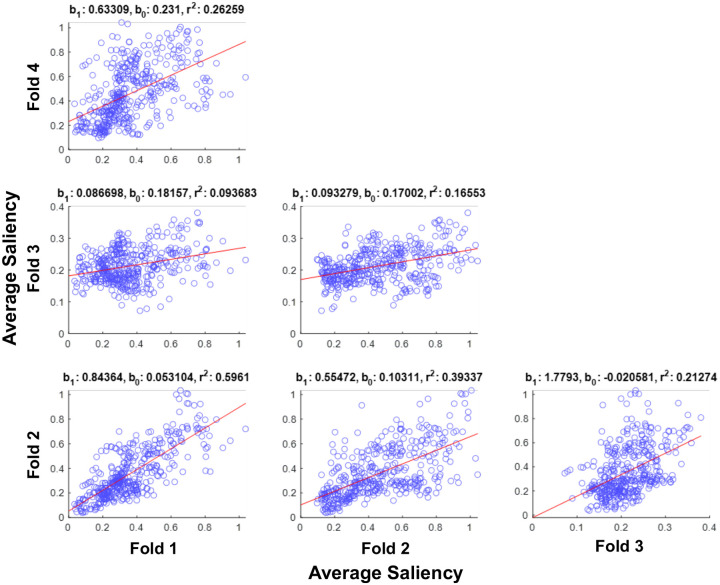
Linear regressions of area average saliency between all folds of the proposed Cutnet classifier model. Each blue point per scatterplot represents an atlas area plotted by average saliency from different folds as aligned on the x and y axes. Fold 3 area saliency averages did not align well with other folds compared to the others. b_1_ and b_0_ represent slope and y-intercept of the regression line (red) respectively. For comparison, a perfect linear alignment for area saliency between different folds is b_1_ = 1 and b_0_ = 0.

Saliency data across subjects in our dataset were generated from different network folds, therefore, it was critical to assess the level of similarity for saliency across folds. As such, we calculated average area saliencies per area (across subjects) per fold and performed a linear regression to observe how mean area-specific saliency scores aligned between folds. We were able to establish that each of our networks’ saliency values reflected similar representations of PD despite being trained on different folds of data (Fig 5).

### Saliency Analysis:

To establish what saliency scores might mean anatomically, we conducted a variety of analysis on the raw saliency scores and established associations with other subject data we had access to through PPMI. Firstly, we established class specific profiles of saliency. We observed that incorrectly classified subjects’ saliency maps mirrored the incorrect class, and therefore we excluded 12/100 incorrectly classified PD subjects and 18/100 incorrectly classified CT subjects from analysis to prevent cross contamination.

Then we examined which areas had the highest raw average saliency for the PD class. This provided a view of which areas stood out most to the network as associated with PD classification. We manually segmented Julich atlas areas into lobes to provide coarser, more interpretable lobe-averaged saliency profiles than the 414 individual atlas areas.

Pairwise area correlation was also analyzed for each network. Kendall’s tau rank-based correlation was calculated over all 88 subjects’ data, resulting in a 414 by 414 correlation matrix indicating how each area’s saliency changed with the saliency of other areas. All insignificant correlations (p > 0.05) were excluded from analysis. Given the possible impact of PD pathology on pathways/functional networks, we segmented our atlas into canonical functional networks mentioned in literature (dorsal attention, ventral attention, executive/frontoparietal, visual, limbic, default mode)^35–37^. Due to their involvement of key PD ROIs like the SNc, we also included nigrostriatal and mesocorticolimbic pathways in this analysis^38,39^. We then used these segmentations to calculate average correlation of areas making up functional networks with other areas in their same networks compared to other areas brain wide. Supp Data 2 contains a full list of areas making up functional network segmentations. To summarize which areas tended to be most central to changes in saliencies of other areas (pairwise saliency correlation between areas), we calculated an area-specific “node score” (*NS*) as presented in Eq 3:

(3) $$NS=\sum_{b}\tau_{a,b}\left(p<0.05\right)$$

Where *a* is a given atlas area, *b* is the set of all atlas areas excluding *a*, *τ*_*a,b*_ between a pair of areas represents significant (p < 0.05) Kendall’s rank-based correlation coefficient. Because of the implicit strength-based weighting of the correlation coefficient, the node score indicates an area saliency’s cumulative tendency to change with the saliency of others, meaning that high node score areas may form central nodes in networks of saliency.

Finally, we examined Pearson and Kendall correlation between area saliency measures and subjects’ score from UPDRS P3, MoCA, LEDD and time since first symptom reported. If a significant correlation existed, it provided evidence that the network’s representation of PD was reflective of clinical measures that had independently been shown to be sensitive to PD progression. This also allowed us to identify which specific atlas area saliencies were more correlated to changes in motor and cognitive scores.

## Results:

### The Classifier Model

After k-fold training, the Cutnet model was able to achieve an average classification accuracy of 84.5% ± 7.95% across the four folds, F1–4 (precision: 0.82 ± 0.13, recall: 0.87 ± 0.095, F1: 0.84 ± 0.063). Table 1 shows F2 was the most accurate during testing at 90%, while F3 was least accurate at 78%, overfitting its training partition. Given the limited dataset size, it is likely this partition of training subjects was less informative for accurate classification of its testing set. All other folds’ testing accuracy better fits their training partition.

Linear regression analysis between all four folds of the network revealed correlations between all folds. Correlations involving fold 3 have slopes furthest from 1 (Fig 5: F1 vs F3: b_1_ = 0.086698, F2 vs F3: b_1_ = 0.093279, F3 vs F4: b_1_ = 1.7793). Fold 3 also overfit and thus had a lower accuracy than the other 3 folds (Table 1). For these reasons, exclusion of fold 3 from saliency analysis was considered. Examination of saliency correlations with subject data (UPDRS P3, MoCA) per fold indicated that fold 3 aligned with the findings of the others, and thus fold 3’s test subjects were included as they did not constitute true outliers in other methods of analysis.

12 of the 100 PD subjects were misclassified by the network. All misclassified subjects were considered early onset even for this predominantly early PD dataset (mean: 2.32 ± 0.54 years since first symptoms) and were all right-handed. All but one were unmedicated, with the sole on-medication subject having had symptoms the second longest among the misclassified group.

### Raw Saliencies:

**Figure 6:**
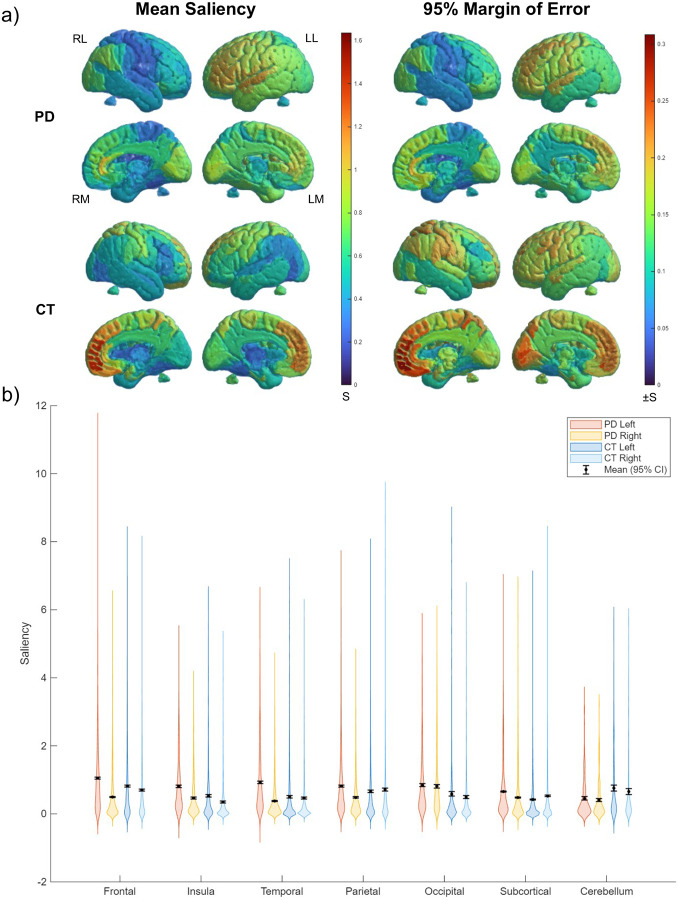
Average saliency score profiles between CT and PD classes. a) The top row of plots corresponds to the mean saliency per area across all Parkinson’s Disease (PD) subjects, while the bottom row depicts mean saliencies for control (CT) subjects. The right column displays the 95% margin of error for each class across subject average (rows). Views labeled as follows: RL (right lateral), LL (left lateral), RM (right lateral), LM (left medial). b) Saliency distribution for areas organized in lobes split by hemisphere across CT and PD subjects. Group mean saliency with 95% CI is imposed over violin distributions in black.

Averaging the area saliencies across all PD and CT subjects respectively showed distinct saliency profiles between the two classes, as displayed in figure 6. The highest saliency areas for the PD class (Fig 6a, top) were left lateralized, with the strongest saliency in the left frontal lobe (mean: 1.0495 ± 0.0286, higher than all other regions). Left lateralization was observed in all lobes except the occipital, which had overlap between left and right 95% CIs (mean (R): 0.8107 ± 0.0476; mean (L): 0.8430 ± 0.0481, Fig 6b). Many atlas areas in the left insula as well as the superior temporal lobe showed high average saliency (Fig 6a, around 1.3). The occipital lobe had substantially higher saliency in the right hemisphere than all other right hemisphere lobes for PD. While slightly lower magnitude, parcellations of the left orbitofrontal cortex (OFC) and the left anterior cingulate cortex (ACC) showed high average saliency (Supp Data 3). The CT saliency profile was different than that of PD. It was more bilateral and was highest for areas around the medial parts of the occipital and frontal lobes. For both classes, subject saliency distributions tended to be long tailed for areas that were higher in mean saliency (Fig 6b).

### Pairwise Correlation & Functional Networks

**Figure 7:**
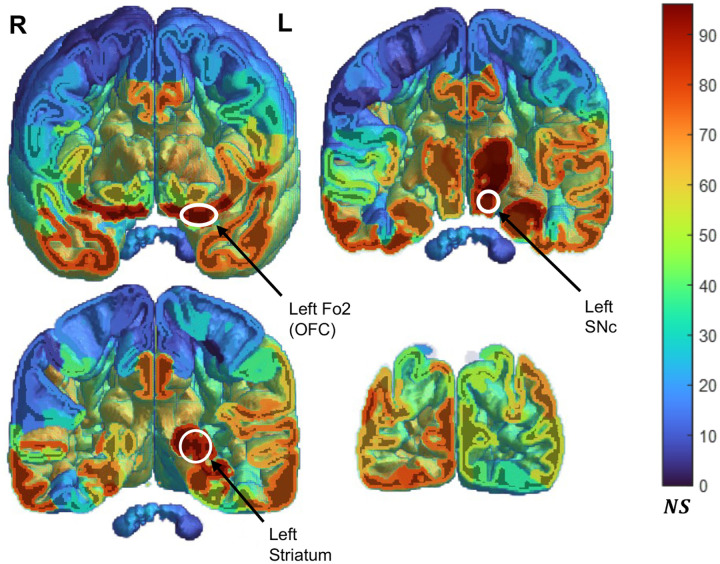
Area node score volume depicting the node score of each atlas area. A given area’s node score is the sum of tau coefficients from significant correlations with all other areas. A higher node score indicates an area’s saliency is strongly correlated with the saliencies of many of the other 413 areas. Cooler areas mean a lower node score, where warmer colors indicate high node score. Slices progress from anterior (top left) to posterior (bottom right), viewed from supine position (L=left hemisphere, R=right hemisphere). Striatum (basal ganglia), SNc, and orbitofrontal cortex (OFC) atlas ROIs are labeled.

Calculating pairwise areal saliency correlations revealed a different profile of underlying connectivity compared to raw score averages. Correlations were visualized via area specific node score, which was the sum of the Kendall’s tau coefficients for significant correlations with other areas. This gave a coarse picture of how much an area’s saliency was associated with all others brain wide. Despite ubiquitously lower raw saliency scores, subcortical areas such as the SNc and other ROIs like the OFC and basal ganglia had high node scores, Where the entire left frontal lobe exhibited high average saliency, regions that made it up had lower node scores, apart from areas nearer to the insula such as the left frontal operculum (Fig 7). The full pairwise correlation matrix and associated p-values are available in Supp Data 4 & 5.

While node score provided a succinct metric to measure the tendency of an area to associate in networks of saliency brain-wide, it did not provide direct information on exactly which pairs of areas were highly correlated. To frame this analysis, we organized areas into canonical functional networks to see if saliency scores from areas within these networks tended to be stronger correlated with one another (intra-functional network), or if our pairwise saliency correlations appeared to be agnostic to these functional connections^35–37^.

**Figure 8:**
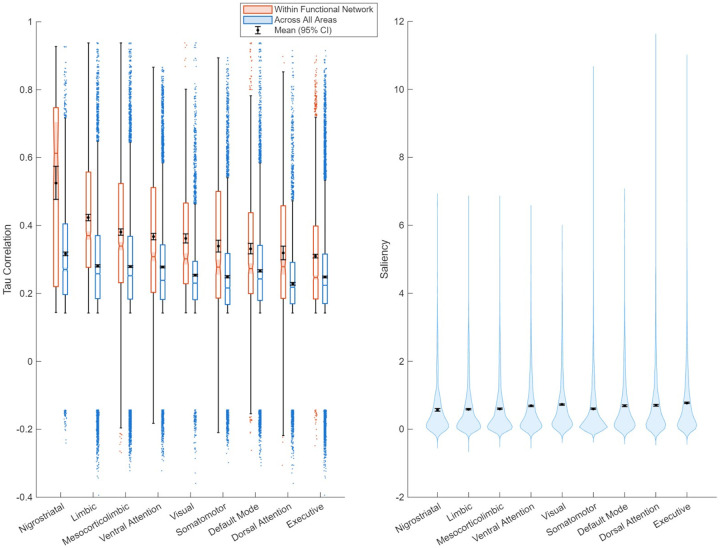
Functional networks are distinguished by pairwise saliency correlation, but not by raw saliency. Left: average pairwise saliency correlation for an area with other areas within functional network compared with pairwise saliency across all other areas regardless of functional network membership. All functional networks had a significantly higher average correlation coefficient among themselves compared to among all brain areas for each network segmented. Right: average area saliency distribution within each of the functional networks. All functional network area saliencies were similarly distributed and did not differ substantially. A complete list of functional area segmentations is available in Supp Data 2.

Figure 8 revealed stronger correlations between areas within functional networks relative to correlation with all brain areas, not just those within the network. Areas within the nigrostriatal pathway, consisting of the SNc, basal ganglia, and subthalamic nucleus (STN), correlated the strongest within-network (average τ coefficient within-network: 0.5254, all areas: 0.3170, non-overlapping CI). The magnitude of difference was not as large for all functional networks, but even very distributed networks such as the DMN associated within itself more than across all areas. Notably, despite their comparatively higher degree of correlation, the nigrostriatal, limbic, and mesocorticolimbic networks/pathways were lower in raw average saliency than the DMN, VAN, DAN, and visual networks (Fig 8, right). The executive/frontoparietal networks were higher saliency on average than all other networks, likely due to high raw saliency of left frontal regions. Overall, pairwise correlation distinguished functional networks while raw saliency did not.

### Associations with Subject Metrics

**Figure 9:**
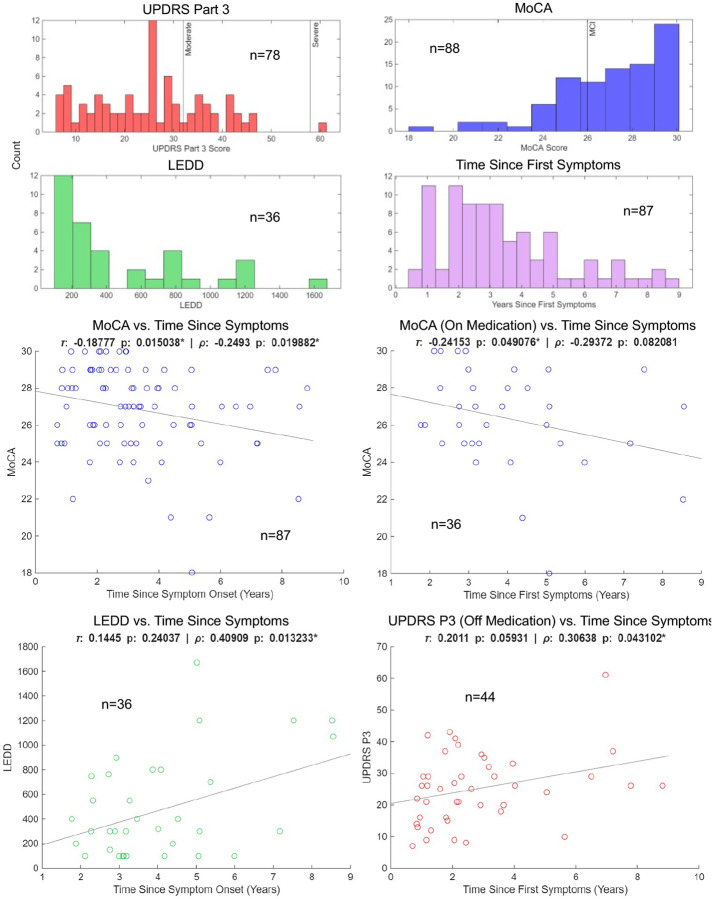
Distributions and associations of subject data. Histogram distributions for cognitive scores (MoCA), motor scores (UPDRS P3), years since first PD symptom (YO), and LEDD are shown on the left. Scores lower than 26 note mild cognitive impairment (MCI) on the MoCA scale^40^. A UPDRS P3 score of 32 or higher is moderate and 58 or higher is severe^41^. Pairwise correlation between subject metrics with at least one significant correlation (p < 0.05*) via Pearson (ρ) or Kendall (τ) are plotted on the right (YO vs MoCA: p_τ_ = 0.015038* p_ρ_ = 0.019882*; YO vs LEDD: p_τ_ = 0.24037 p_ρ_ = 0.013233*; YO vs MoCA on medication: p_τ_ = 0.049076* p_ρ_ = 0.082081; YO vs UPDRS P3 off medication: p_τ_ = 0.05931 p_ρ_ = 0.043102*). All other metrics, including UPDRS P3 on/off and MoCA on/off, were not significantly associated with one another. The number of data points is imposed on all plots and histogram distributions. 88 total PD subjects were included in analysis.

Having established patterns in both raw saliency scores and pairwise area correlations, we then sought to ground our abstract saliency results to concrete subject data sourced from PPMI. Figure 9 depicts the distributions of the four metrics used to measure correlations with area saliency across all subjects in the dataset.

Examining figure 9, the entire PD cohort is considered early progression as defined by Claasen et al. 2016 (< 10 years since symptom onset). Most medicated subjects were on lower doses of levodopa/equivalents (23/36 subjects were < 450 LEDD). Subjects were predominantly not cognitively impaired according to MoCA (64/88 subjects scored > 25), and most experienced lower than moderate motor symptoms according to UPDRS P3 (56/78 subjects < 32)^40,41^. Only one of our subjects had a UPDRS P3 score considered severe (> 52). Of the 12 misclassified subjects, 11 were off medication with the singular medicated subject having LEDD of 400. There were 4 subjects meeting the mild cognitive impairment threshold (MoCA < 26) and 3 subjects with UPDRS P3 above 32, indicating moderate motor impairment. All misclassified subjects were right-handed.

Despite our subject data being skewed to less severe symptoms and earlier PD, we still identified strong correlations between saliency and UPDRS P3/MoCA for many brain regions and PD ROIs.

**Figure 10:**
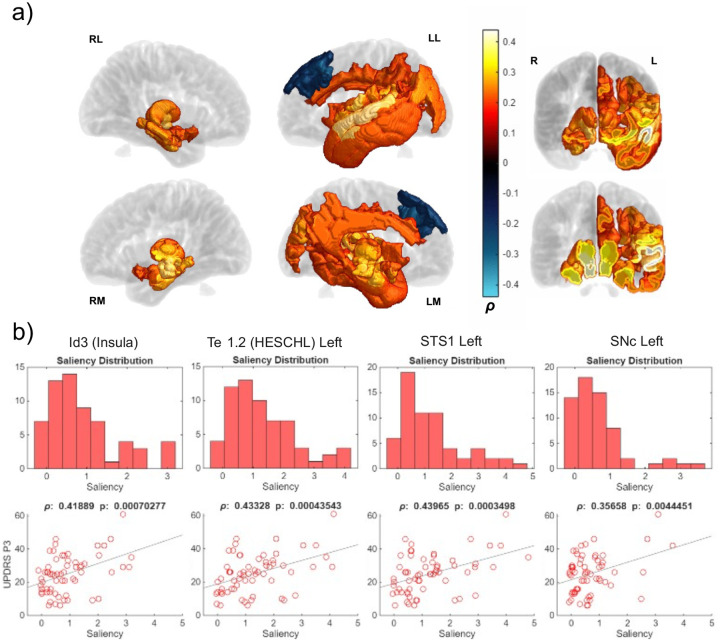
Correlation between UPDRS P3 scores and region-specific saliencies across PD subject cohort. a) UPDRS volumes show regions with significant (p < 0.05) correlations colored according to their Pearson’s rho coefficient (ρ). Areas without significant correlation are shown in transparent gray. Hot colors indicate positive correlations while cool colors indicate negative correlations. Surfaces are labeled by RL (right lateral), LL (left lateral), RM (right medial), LM (left medial). R and L denote right and left hemispheres respectively for coronal slices. b) Area saliency distribution and saliency vs. UPDRS P3 correlation scatterplots for several atlas ROIs (STS: superior temporal sulcus, SNc: substantia nigra pars compacta).

Overall, UPDRS P3 correlated positively with many areas (Fig 10). Saliency scores in the left temporal cortex tended to increase with subject UPDRS P3 scores. Many parcellations of the left insula were also correlated with UPDRS P3 (*ρ* > 0.35). Many limbic areas were correlated bilaterally, including the SNc and SNr, the basal forebrain, many thalamic nuclei, the hippocampus, and the amygdala. Some areas of the medial frontal gyrus/superior frontal gyrus contained weaker negative correlations. Supp Data 6 shows all areas with significant correlations (both Kendall’s tau and Pearson’s rho with p-values).

**Figure 11:**
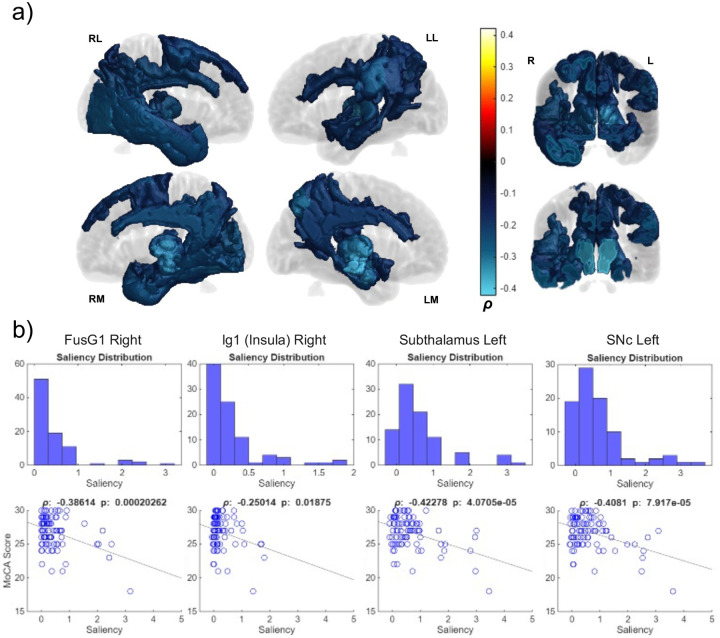
Correlation between MoCA scores and region-specific saliencies across PD subject cohort. A) MoCA volumes show regions with significant (p < 0.05) correlations colored according to their Pearson’s rho coefficient (ρ). All other regions lacked significant correlation and are colored transparent gray. Cool colors indicate negative correlation. Surface labels are as follows: RL (right lateral), LL (left lateral), RM (right medial), LM (left medial). R and L denote right and left hemispheres respectively for coronal slices. B) Four representative ROI’s subject saliency distributions and saliency vs. MoCA correlation scatterplots (FusG: fusiform gyrus, SNc: substantia nigra pars compacta).

MoCA scores correlated negatively with many regions, but higher magnitude correlations (ρ < −0.35) appeared in bilateral limbic and nigrostriatal areas (Fig 11). Much like with UPDRS P3 scores, the temporal regions were correlated with MoCA. However, lateralization of correlation flipped from the left to the right for cognitive scores versus motor scores. Parts of the parietal lobe had weaker negative correlations with a slight left lateralization. Supp Data 7 has a full list of MoCA area correlations (both Kendall’s tau and Pearson’s rho with p-values).

**Figure 12:**
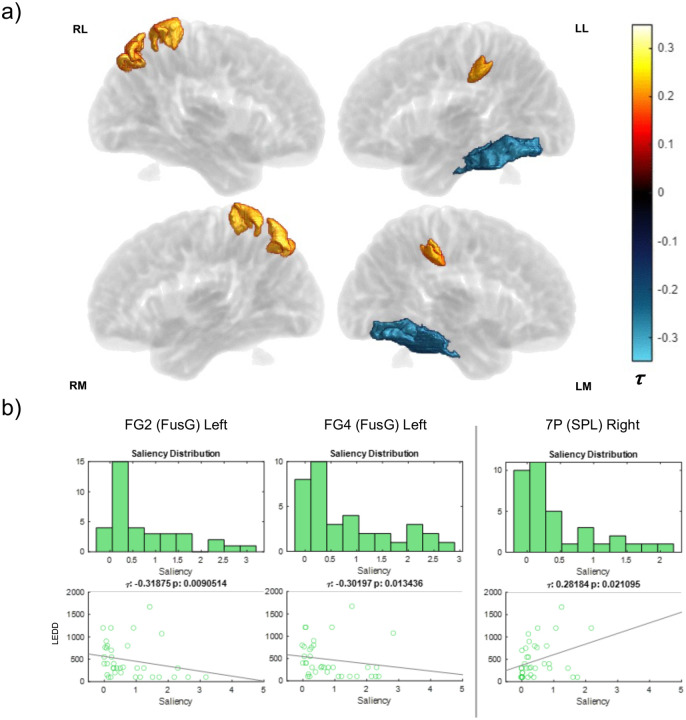
Correlation between Levodopa Equivalent Daily Dose (LEDD) and region-specific saliences across PD subject cohort. A larger LEDD measure means a subject was on a higher dose of DA precursor medication. Because there were fewer medicated subjects than other metrics (N=36), Kendall’s tau (τ) was used to reduce the impact of outliers. a) LEDD volumes depict magnitude of significant Kendall correlations (p < 0.05) colored according to tau coefficient. Regions without significant correlation in transparent gray. Surface labels are as follows (top left): RL (right lateral), LL (left lateral), RM (right medial), LM (left medial). b) Areas with highest magnitude of correlation are shown with saliency distribution and subject scatterplots vs LEDD scores (FusG: fusiform gyrus, SPL: superior parietal lobule).

Instead of mostly uniform positive or negative correlations like UPDRS P3 or MoCA, LEDD had two weak clusters of correlation with few areas and opposite signs (Fig 12). The left fusiform gyrus negatively correlated, while parcellations of Brodmann’s areas 5 and 7 positively correlated in the right hemisphere. Correlations may have been sparse in part because of the limited number of subjects on medication (n = 36) compared to other subject metrics correlated with area saliency data. Supp Data 8 shows a full list of the limited significant correlations with LEDD (both Kendall’s tau and Pearson’s rho with p-values).

**Figure 13:**
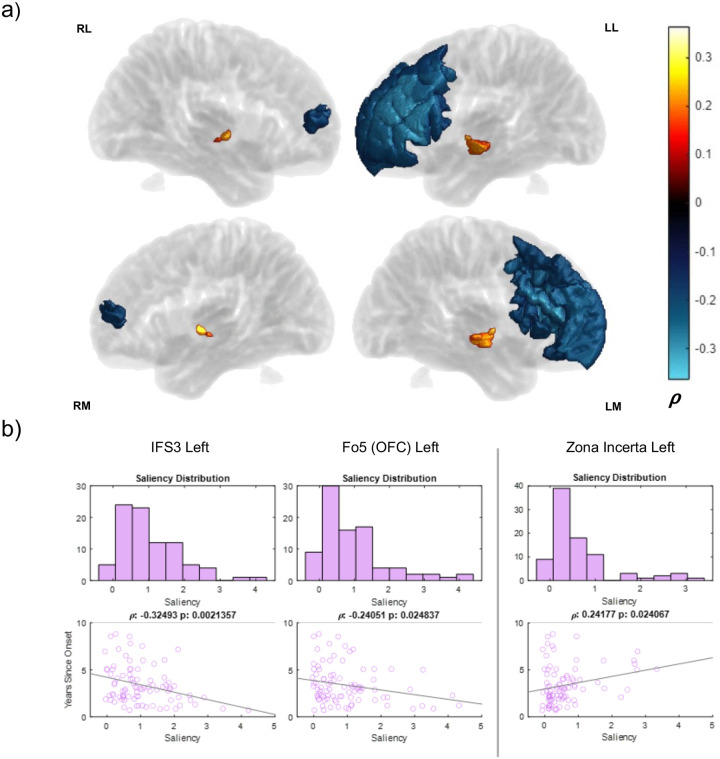
Correlation between years since symptom onset and area saliencies across PD subject cohort. a) Years since symptom onset volumes depict regions significantly correlated time since symptom onset colored by magnitude of significant Pearson correlations (p < 0.05). Regions without significant correlations are in transparent gray. Surface labels are as follows (top): RL (right lateral), LL (left lateral), RM (right medial), LM (left medial). b) Saliency distribution and subject scatterplots vs time since PD symptoms for representative ROIs reflecting two clusters of correlation in volumes. These plots are separated by negative frontal correlations on the left and positive limbic correlations on the right (IFS: inferior frontal sulcus, OFC: orbitofrontal cortex).

There were two distinct parts of the brain with opposite correlations against time since PD symptoms were first experienced (Fig 13). Most left frontal regions correlated negatively, showing that this region’s saliency tended to be lower in subjects who had had PD for longer. A small cluster of left-skewed limbic regions including various thalamic nuclei and the STN were positively correlated with time since onset of symptoms. Our cross-sectional dataset represented some coarse regularity to saliency profile over time, with saliency being more frontal early in PD progression to more limbic/thalamic regions later. See Supp Data 9 for a full list of correlated areas.

## Discussion:

Through patterns in raw saliency and its correlations with cognitive and motor scores, we find that atrophy is likely a robust feature contributing to regional saliency scores. Furthermore, pairwise correlation between area saliency scores linked areas known to be functionally connected. Correlations within functional networks were greatest within the nigrostriatal pathway and limbic network, showing PD impacts these connected areas collectively. This supports theories of specific network-level effects of PD neuropathology, particularly within dopaminergic pathways. Saliency captured PD progression along multiple axes of symptoms and disease characteristics, as revealed by wide correlations between saliency scores and UPDRS P3, MoCA scores, and number of years since symptom onset. Bilateral limbic and nigrostriatal saliencies were best connected to motor and cognitive scores. Our findings suggest these areas and networks play a central role in driving both motor and cognitive decline. Areas within the frontal left hemisphere did not have many correlations with either MoCA or UPDRS P3 but negatively correlated with the time since symptom onset. Despite not being as correlated to PD symptoms, the left frontal hemisphere could still contain cortical markers of early PD.

### Saliency Could Indicate Atrophy

We successfully extracted comprehensive quantifications of area specific saliency data from a “black box” 3D CNN model. We next sought to understand what the saliency output means and why it could be informative. Fundamentally, our area specific saliency score quantified how much our network perceived regional discriminatory importance between control and PD. It did not, however, explicitly define what those differences were beyond the fact that they were consequential to the network’s classification of a subject. A given area may have been salient for PD because it showed atrophy relative to control, but this was an inference that we clarified by comparing saliency with cortical thickness findings from literature and through establishing a connection between saliency and subject attributes associated with atrophy, like cognitive scores and motor performance assessments.

Cortical thickness measurements on MRI datasets done using tools like Freesurfer have demonstrated association between atrophy in PD and declining cognitive scores^13^. In our data, saliency followed a similar pattern, as declining cognitive scores correlated with saliency for 152/414 areas. Areas with highest raw saliency in our data mirrored areas with highest gray matter volume reduction in early PD from Claassen et al. 2016. This study measured differences in atrophy between a late and early group of PD subjects, revealing a left hemisphere-biased profile of atrophy in the frontal lobe and insula in early PD which progressively increased bilaterally and toward posterior areas in later PD subjects (over 10 years). While our dataset was composed entirely of subjects considered early PD, we found elements of both early and late profiles in our raw saliency output. Saliency was left lateralized in the frontal lobe but became progressively less laterally biased in posterior regions (Fig 6). Our saliency data reflected findings from cortical thickness studies through raw saliency and its correlations with motor and cognitive symptoms (Fig 10, Fig 11 respectively), supporting the hypothesis that atrophy is likely a robust feature that contributes to network saliency scores. It is also unlikely to have been the only feature captured by saliency. Saliency is a high dimensional metric and could have captured subtle structural changes connected to changes in connectivity, or other pathological features separate from atrophy.

While saliency does not explicitly measure any specific feature, it will identify any structural change that discriminates PD MRIs from CT MRIs in any of white matter, gray matter, brain stem, etc. Where a measure like cortical thickness will ignore changes in white matter hyperintensity, for example, our 3D CNN will capture those changes if they help the network distinguish between classes. This opens multiple possibilities for different ways to segment or atlas saliency maps. As white matter atlases improve and become more popular, saliency could be a generalizable tool to apply to more than only gray matter segmentation templates and atlases^42^.

### Saliency Reveals Network Level Relationships in Structural Imaging

A well-trained CNN could be sensitive to a variety of features indicative of PD and reveal which features appear on a per-subject basis through GradCAM reflective of PD pathology for that specific individual. Long-tailed distributions for high saliency left frontal and insular regions indicated that our saliency scores reflected the high degree of subjectivity CNNs can account for (Fig 6). To respect the heterogeneity that existed within our data, we analyzed how different areas’ saliencies change with one another independent of the magnitude of saliency through pairwise area correlation.

Despite their relatively weaker raw saliency, we observed that subcortical areas were heavily associated brain-wide via rank-based pairwise correlation as interpreted by node score (Fig 7). We then established specific patterns of correlation using functional networks as a frame, with an eye on subcortical areas participating in dopaminergic pathways heavily implicated in PD pathology. This also provided insight into whether our 3D CNN could pick up trends in structural images rooted in underlying functional connectivity. Saliencies of areas within all segmented functional networks associated with one another considerably more than they associated with all other brain areas agnostic of functional connectivity, revealing that saliency changes tend to occur over networks and pathways of functionally connected areas (Fig 8). The differing magnitudes between intra-network and all-area (functional network agnostic) correlation corresponded with PD pathology. The heavily implicated nigrostriatal pathway, which includes the SNc, Substantia Nigra pars reticulata (SNr), striatum, and the STN, showed the greatest differential in within-network vs all area correlation (Fig 8a, 0.208 difference in τ magnitude). This correlation differentiated this pathway, which was otherwise lowest in raw saliency (Fig 8b). The weakest associated functional network, the executive (or frontoparietal), was higher in average saliency than all others. These findings show that saliency captured important second order relationships between areas otherwise invisible via raw saliency. In addition, cross-area correlation analysis proves saliency is linked across areas with functional connectivity. Furthermore, the magnitude of the differential in correlation reflecting PD specific nigrostriatal pathology. More generally, our model capturing PD specific network associations shows that 3D CNNs are effective tools for parsing functional connectivity from structural data. 2D CNNs would not be able to replicate these findings, as functional connectivity exists in 3D space within the brain and hence necessitates volumetric processing.

### Medication Use Distinguished Misclassified Subjects

Examining the 12 misclassified subjects of our PD cohort, we observed all were relatively early cases in our dataset (only 2 subjects were above 3 years since symptoms were reported, all were less than 4 years). The UPDRS P3 and MoCA score distributions of these 12 subjects match that of the full dataset distribution. All 12 misclassified subjects were right-handed. While none of these metrics substantially differentiated this cohort from the entire dataset, their medication use constituted an outlier, as only one was medicated (8.33%) with an LEDD of 400 compared to 36% being medicated over the total PD cohort. The network performing better on medicated subjects could mean that a subject being on medication indicates more progressed stage of pathology, perhaps as a reactive measure. Correlations between subject saliency data and LEDD suggested there were weakly connected features to amount of medication used in BA7 and in the left fusiform gyrus among correctly classified subjects (Fig 12). There were, however, fewer medicated subjects (n=36), potentially making it difficult to observe a robust relationship.

### Relationships Between Symptoms and Medication/Length of PD

To further clarify the relationships between symptoms and medication/time on PD symptom progression, we examined correlations between all non-imaging metrics in the dataset. While we did not observe significant correlation between MoCA, UPDRS P3 nor LEDD in our dataset, both motor symptoms and cognitive symptoms are known to be influenced by levodopa or DA agonist use^43,44^. Levodopa response is not uniform across subjects, and it’s possible its efficacy varied highly in our dataset such that no clear correlation was discerned^43^. It is also important to consider the cross-sectional nature of our dataset, as medication use/dosage may increase in reaction to worsening motor or cognitive performance. If this is the case, a longitudinal view where reactive increases in LEDD in follow-ups are observable could lead to clearer picture of how LEDD influences PD progression. While the pattern of LEDD increasing over time is apparent in our dataset (LEDD correlated with time since symptoms, Fig 9), LEDD’s did not correlate with motor and cognitive scores and had sparse association with area saliency scores from the network. This could have been a result of the cross-sectional nature of our data and lower number of medicated subjects (Fig 12).

Medication did seem to play a role in aligning symptom progression with time since first PD symptoms, however. Off-medication UPDRS P3 scores significantly correlated with time (p_ρ_ = 0.0431*; p_τ_ = 0.0593) while on-medication UPDRS P3 did not, indicating medication influenced motor symptom progression over the disease time course. This finding is corroborated by other studies, which have found UPDRS P3 correlates better with time over 5-year pools for off-medication subjects than on-medication^41^. MoCA, independent of medication status, significantly correlated with time since symptoms (ρ = −249, p_ρ_ = 0.0199*; τ = −0.188, p_τ_ = 0.015038*; n = 87). There still appeared an influence of medication, however, as correlation was significant and stronger for on-medication MoCA (ρ = −0.294, p_ρ_ = 0.082; τ = −0.242, p_τ_ = 0.0491*; n = 36) while off-medication MoCA did not significantly correlate with time since symptoms. This could mean medication stabilizes cognitive decline on a longer time scale, reducing intersubject variability in the development of PD related cognitive deficits.

### Different Regions Correlated with Motor Symptoms, Cognitive Symptoms, and Length of PD

To connect saliency to UPDRS P3 and MoCA, we correlated regional saliency with subject scores to establish localized profiles of association. Many regions correlated with MoCA and with UPDRS P3, while time since onset correlated with a sparser set of areas, implying saliency may better reflect motor and cognitive symptoms.

Saliency’s predominantly positive correlation with UPDRS P3 supports the hypothesis that saliency accounts for atrophy (UPDRS P3 score increases as motor symptoms worsen, and increased saliency could imply atrophy underpinning motor difficulties). Most subcortical areas correlated with UPDRS P3 across both hemispheres. This finding recapitulated other literature reflecting correlations between motor symptoms and decreased volume in the SNc, SNr, and striatum^4,45^. In addition to subcortical areas, the left insula and left temporal lobe also correlated strongly with UPDRS P3 (Fig 10). This hemispheric bias was perhaps due to our overwhelmingly right-handed PD cohort (97%), as the left hemisphere is more motor dominant in nature in right-handers^46^. Following this hypothesis, left-handed subjects may have had right lateralized saliency profiles that were washed out in averaging due to their being 3 datapoints out of 88 in our dataset. After manually examining the GradCAM saliency maps from left-handed subjects, we did not observe distinct right-lateralized saliency profiles. This did not disprove the hypothesis that left lateralization in motor score saliency correlation was due to a right-handed subject bias, however, as this small sample of subjects may have had bilateral atrophy the network ignored in favor of telltale PD features in its preferred left hemisphere. This bias likely naturally existed in the network due to the predominantly right-handed training set. Saliency was not universally positively correlated with UPDRS P3, as several regions in the left frontal lobe negatively associated with UPDRS P3 score. This correlation was lower in magnitude than most of the positive correlations but indicated that atrophy was not the only feature giving way to saliency. The network may have been able to identify other abnormalities in cortex connected to fewer motor symptoms in these regions for several subjects.

Like UPDRS P3, the MoCA saliency profile includes bilateral subcortical correlation, however, temporal regions were right hemisphere biased as opposed to left (Fig 11). Right hemisphere saliency may have indicated more progressed and/or longer-term PD cases, despite none of the right hemisphere and only sparse thalamic regions correlating positively with time since symptom onset. In addition to MoCA scores correlating negatively with right temporal saliency, subject MoCA scores correlated with time of disease within our dataset. It has been observed that the risk of dementia increases substantially in long term PD (3–12% risk at year 5 to 74% at year 20)^47^, further connecting PD time course with intersubject cognitive decline. Both right temporal lobe saliency and time since symptom onset correlated independently with MoCA score, while no right temporal regions correlated with time since symptom onset alone. This may mean that right (or bilateral) hemisphere saliency in progressed cases may have been better connected to cognitive decline than left hemisphere saliency but was more heterogeneous than the more homogenous left frontal lateralization in early cases observed by in our data and by Claasen et al. 2016 (Fig 13).

Despite there being a temporal aspect to development of PD-dementia in literature and in our data (Fig 9), it seems time does not independently characterize or predict PD progression on a fine timescale and is rather a more coarse predictor. Based on the characterization of early atrophy being left frontal biased, negative correlation between time and saliency implies that saliency can decrease (or be “stolen”) in favor of a more distributed set of areas showing changes in later cases (Fig 12). There was little overlap between areas correlating with PD time course and cognitive/motor scores, despite MoCA and UPDRS P3 (unmedicated) correlating with time independently (Fig 9). These findings indicate that temporal patterns of PD pathology are weaker on a fine scale and somewhat decoupled from the severity of specific symptoms on an independent, per subject basis. Instead, in some regions, saliency scores were more robustly correlated with symptoms as measured by UPDRS P3 and MoCA. We may have observed more robust, brain-wide correlations with a coarser pooling of temporal data, accounting for high intersubject variability in the time course of PD pathology.

### Limitations:

While our network was capable of performance comparable to clinical PD diagnostic accuracy, it is important to consider the effect of the limited dataset on the model’s representation of PD. It is unlikely our dataset of 100 PD and 100 CT MRI volumes captured a complete profile of the heterogeneous PD-associated structural features available in T1w MRI. The model would have benefitted from more MRI data from PPMI to further increase performance on the difficult task of classifying early PD.

Methods of preprocessing may have also introduced small errors. The atlasing approach relied on registering all subjects to the same anatomical space to align Julich Brain parcellations with subject data. It is therefore impossible for the atlas to be perfectly accurate subject to subject, but this is accounted for in part by probabilistic parcellations, where each image voxel has a probability of belonging to an atlas area instead of a binary assignment. This helps normalize the influence of anatomical variability between subjects regardless of registration. Nevertheless, there were still small errors with intersubject atlas alignment. Innovative deep-learning-based per subject segmentation techniques could be employed to increase per subject accuracy, provided error is considered and validated^48^.

To align the atlas, all MRIs had to be registered to MNI152 space. This could have created bias when maintaining accurate representations of all brain regions across subjects, as they were all morphed and transformed during the registration process to fit the template anatomical space^49^. This could be especially impactful when registering MRIs of subjects with PD pathology, as areas change in ways which may influence how they are registered compared to control brains^50^. PD-based anatomical spaces such as PD25 exist and could have been the template chosen for spatial normalization, but they are not natively compatible with the Julich Brain Atlas. While the atlas could theoretically be registered to another anatomical space, this process has not been validated, and controls would also have needed to be registered to a pathological anatomical space.

### Conclusion & Future Work:

In this study, we propose a 3D CNN capable of classifying PD in T1w MRIs and introduce a generalizable, powerful, and robust explainability method to interpret these traditionally opaque models in a way that can be thoroughly quantitatively analyzed. We paved the way for analyzing the diverse set of features this metric contains by showing it could be connected to cortical thickness, and that it correlated functional networks through structural data alone. We also connected saliency scores of predominantly subcortical and temporal regions with both motor performance and cognition via UPDRS P3 and MoCA. This study should be repeated on a larger cohort of PD subjects from PPMI, with cortical thickness analysis performed on the same dataset used to train the 3D CNN model to allow for direct connection between atrophy and saliency. This study could also be replicated for detection of any variety of pathologies where large quantities of MRI data are available using any 3D CNN architecture, as GradCAM is generalizable^31^. Within PPMI data alone, prodromal MRIs could be added to the dataset to establish anatomical differences that exist between control, prodromal, and PD. More work can also be done to clarify features captured by saliency. Saliency could also be compared with any subject metric, not just the few used in this study (UPDRS P3, MoCA, time since symptom onset, LEDD). Saliency is a complex metric that is influenced by a variety of features and information from training MRIs. Linkages and associations can be investigated and clarified by examining its connection to a multitude of metrics.

## Supplementary Material

Supplement 1

Supplement 2

## Figures and Tables

**Table 1:** Per fold and overall Cutnet performance on the binary PD classification task. Statistics were calculated from the folds’ performance on their 50-volume testing partitions, then all folds were averaged (including 95% confidence interval). All folds’ testing GradCAM data were ensembled to include unseen saliency data for each subject in the dataset.

Fold	Accuracy	Precision	Recall	F1 Score
**F1**	84%	0.75	0.9545	0.84
**F2**	90%	0.90	0.8571	0.8780
**F3 (Overfit)**	78%	0.75	0.84	0.7950
**F4**	86%	0.875	0.84	0.8571
**All (95% CI)**	84.5% (± 7.95%)	0.82 (± 0.13)	0.87 (± 0.095)	0.84 (± 0.063)

## Data Availability

Data used in the preparation of this article was obtained on 2024-06-20 from the Parkinson’s Progression Markers Initiative (PPMI) database (www.ppmi-info.org/access-dataspecimens/download-data), RRID:SCR_006431. For up-to-date information on the study, visit www.ppmi-info.org. Statistical atlas parcellations were retrieved on 2025-06-17 from the Julich Brain Cytoarchitectonic Atlas v3.1 using the Siibra Python Package^34^.
