# Supplementary material for: Explainable 3D CNNs link regional and network level disruption in early Parkinson’s MRIs to symptom progression
Source: bioRxiv. 2025 Dec 3:2025.11.17.688918. Preprint. [Version 2] doi: 10.1101/2025.11.17.688918 (PMC12694578; doi:10.1101/2025.11.17.688918)
Supplement: Supplement 1 [file media-1.pdf]

Explainable 3D CNNs link regional and network level disruption in early Parkinson’s MRIs to symptom progression

Ezekiel Moroze, Basilis Zikopoulos, Arash Yazdanbakhsh

PDMS_SuppData_Final.xlsx

Supplemental Data 1: Full list of areas/regions retrieved from Julich Human Brain Atlas V3.1. There were 414 areas in total.

Supplemental Data 2: List of areas segmented into different functional networks. Atlas areas were manually segmented into functional networks they belong in based on a variety of functional studies and literature.

Supplemental Data 3: Collection of all mean saliency data for CT and PD classes. Saliency is split by hemisphere for each atlas parcellation and colored in accordance with magnitude. Areas are sorted within lobe by highest to lowest PD left hemisphere saliency. 95% confidence margin is next to each mean saliency, with binary computations of CI overlap for within class between hemisphere saliency, and between classes within the same hemisphere saliency. Lobes are organized according to the color key on the right: frontal (light green), insula (pink), temporal (yellow), parietal (light blue), occipital (dark blue), subcortical (orange), cerebellum (light grey).

Supplemental Data 4: Full correlation matrix of rank-based pairwise correlation between saliency scores of all atlas areas.

Supplemental Data 5: p-values corresponding to pairwise correlations (Supp Data 4).

Supplemental Data 6: Full table of area saliency correlations with UPDRS P3 (motor). Both Kendall’s tau (rank-based) and Pearson’s r (parametric) were calculated for thorough evaluation of relationships in the data. All areas shown are significantly correlated ($p < 0.05$).

Supplemental Data 7: Full table of area saliency correlations with MoCA scores (cognitive). Both Kendall’s tau (rank-based) and Pearson’s r (parametric) were calculated for thorough evaluation of relationships in the data. All areas shown are significantly correlated ($p < 0.05$).

Supplemental Data 8: Full table of area saliency correlations with LEDD (medication). Both Kendall's tau (rank-based) and Pearson's r (parametric) were calculated for thorough evaluation of relationships in the data. All areas shown are significantly correlated ($p < 0.05$).

Supplemental Data 9: Full table of area saliency correlations with time since first PD symptoms. Both Kendall's tau (rank-based) and Pearson's r (parametric) were calculated for thorough evaluation of relationships in the data. All areas shown are significantly correlated ($p < 0.05$).
